# Assessment of physical activity in patients with chronic kidney disease and renal replacement therapy

**DOI:** 10.1186/s40064-015-1338-3

**Published:** 2015-09-21

**Authors:** William S. G. Hayhurst, Aimun Ahmed

**Affiliations:** Renal Department, Royal Preston Hospital, Lancashire Teaching Hospitals NHS Foundation Trust, Preston, UK; Medical School, University of Manchester, Manchester, UK; Nephrology Department, Faculty of Medicine, Ain Shams University, Cairo, Egypt

**Keywords:** Physical activity, Human Activity Profile, Chronic kidney disease, Renal replacement therapy, Home haemodialysis, Peritoneal dialysis, In-centre haemodialysis, Questionnaire, Cardiovascular disease

## Abstract

This study aimed to create a unique assessment tool to investigate the physical activity levels of patients suffering from chronic kidney disease (CKD). Previous studies have documented a lack of physical activity within patients living with CKD and patients on renal replacement therapy (RRT). Physical activity plays a key role in the prevention and management of cardiovascular disease (CVD). Patients with CKD are at high risk of developing CVD. Physical inactivity can be considered as a major modifiable risk factor for CVD in CKD patients. In this study 100 patients completed the 20 item questionnaire which produced a total activity score (TAS) and activity loss score for each patient. Bio-chemical markers, haemoglobin, erythropoietin therapy and co-morbidities were documented for each patient. Results were compared against 50 age, sex-matched controls. Results showed a significant difference in TAS between in-centre haemodialysis (ICHD) patients and the healthy control group. A significant difference was also seen with the home haemodialysis (HD) and ICHD patients against the transplant group (TX). Our quick assessment tool identified the ICHD patients as the most physically inactive group within the CKD and RRT population compared to a healthy control group. We therefore recommend that doctors encourage physical activity within this high risk group and start ‘prescribing exercise’ to their patients.

**Electronic supplementary material:**

The online version of this article (doi:10.1186/s40064-015-1338-3) contains supplementary material, which is available to authorized users.

## Background

Chronic kidney disease (CKD) is a worldwide recognized public health issue that affects up to 10 % of the UK population. CKD is associated with cardiovascular morbidity and mortality that places a considerable strain on global health care resources (Meguid El Nahas and Bello 2005). Cardiovascular disease (CVD) is the leading cause of death within the CKD population, with an inverse relationship between a decreasing kidney function and increasing prevalence of CVD (Weir 2011). Due to the ever increasing incidence levels and lack of awareness of the devastating cardiovascular complications associated with CKD, such as premature death, coronary artery disease and cardiac arrhythmias, strategies need to be adopted to minimise the cardiovascular morbidity and mortality in the CKD population (Sarnak 2003).

There is now irrefutable evidence supporting the role of physical activity in the prevention and management of CVD. Within the general population, inactive patients who have increased their physical activity level have shown both increases in functional status and quality of life (Warburton et al. 2006). Recent studies have shown a significant reduction in CVD risk factors associated with physical activity. Physical activity regulates chronic inflammation, oxidative stress, and endothelial dysfunction within the cardiovascular system and has shown health benefits also seen in diabetes mellitus, cancer prevention, obesity, hypertension, osteoporosis and osteoarthritis (Warburton et al. 2006; Steffen-Batey et al. 2000).

Previous literature reported that physical inactivity is common amongst the CKD population and end stage renal disease (ESRD) patients on renal replacement therapy (RRT), even with the compelling evidence that exercise is safe and beneficial in this group of patients (Painter and Marcus 2013; Johansen 2005). Current guidelines recommend a minimum of 1000 kcal of physical activity a week in order to acquire the health benefits seen from exercise. However, literature has suggested that only a 500 kcal extra energy expenditure a week will show health benefits (Paffenbarger et al. 1993). This volume of exercise may seem more appealing to a patient suffering from a chronic illness and therefore encourage previously sedentary patients to become more active. Therefore in order to encourage activity within this high-risk group, the public’s perception of CKD must change from that of a life debilitating illness to one that requires a public health approach for prevention, early detection, and management.

There are a number of questionnaires available to assess physical activity levels. However, the majority of these questionnaires have shown limitations with their reliability and validity (Bastone et al. 2013). From a study carried out by Robinson-Cohen et al. (2013), it was found that the Human Activity Profile (HAP) ‘exhibited enhanced correlation’ to several physical activity scores, including the Physical Activity Scale for the Elderly (PASE), International Physical Activity Long Questionnaire (IPAQ) and the Four Week Physical Activity History Questionnaire (FWH). Another study looking at the validation of physical activity questionnaires in 39 patients on haemodialysis, found that both the HAP and SF-36 questionnaires correlated equally with that of the physical performance measures (Johansen et al. 2001). A major limitation of the HAP questionnaire is the length of time it takes to complete. With over 94-items, this questionnaire can take up to 10 min to complete (Bilek et al. 2005). Studies have also reported that shortening relatively lengthy questionnaires, significantly increases response rates (Sahlqvist et al. 2011). Therefore a new, abbreviated version of this questionnaire is needed to thoroughly assess physical activity within the clinic and community.

This cross sectional study aims to assess the activity level of patients suffering from CKD (non-dialysis) and patients with ESRD on RRT, including patients with a functioning kidney transplant. We will use a self-created, unique, 20 item questionnaire comparing physical activity scores within different CKD and RRT population. We aim to describe where the optimal activity level may lie and where the greatest amount of activity may be lost within the renal population. Furthermore we aim to create an easy assessment tool that can be used within the clinic and community to identify patients at risk of associated CVD within the CKD population.

## Methods

### Subjects

100 patients were asked to fill out the self-created questionnaire before their clinic appointment at Royal Preston Hospital in May–June 2014. Approximately 20 patients from the following groups: CKD stages 3–5 not on any form of RRT, home-haemodialysis (HHD), hospital-haemodialysis (ICHD), peritoneal-dialysis (PD) and transplant (TX) patients were asked to fill out the questionnaire. Verbal consent was gained from each patient prior to filling out the questionnaire. On average each questionnaire took approximately 60 s to fill out.

Inclusion criteria for the CKD group required patients to be >18 years old, not to be on any form of RRT, between stages 3–5 CKD (eGFR <60 mL/min) and known to the renal service for >6 months prior to filling out the questionnaire. Inclusion criteria for the end stage renal disease group on RRT included; >18 years old, on RRT >6 months. For all five groups, patients had to be mobilising without a frame, and not on any formal exercise programme.

### Questionnaire

Two published questionnaires were combined to produce one physical activity questionnaire. The two activity questionnaires were the ‘General Practice Physical Activity Questionnaire’ (GPPAQ) and the ‘Human Activity Profile’ (HAP) (NCC for N and SC 2008; Davidson et al. 2007). The majority of the items and the scoring system were taken from the validated HAP questionnaire. Elements of the GPPAQ were also included to highlight the role of physical activity in the workplace (e.g. if the patient’s job involves vigorous work).

Careful selection of the items was required to enable a thorough evaluation of patient activity levels whilst maintaining the concise nature of the questionnaire. An expert within the renal rehabilitation and exercise medicine field carefully selected questions to incorporate home, work and leisure activities to create an abbreviated questionnaire based on the HAP and GPPAQ. Patients were given 3 response options to each item; (1) they are still doing this activity; (2) they have stopped doing this activity since their CKD diagnosis/started RRT; (3) they never did this activity (therefore has no effect on their score) (Additional file 1).

This method of scoring produced two scores for each patient:

1. The total number of activities that the patient is able to perform—total activity score (TAS).
2. The total number of activities that the patient has now stopped doing since their diagnosis with CKD (for the CKD not on RRT) or since starting their RRT—activity loss score.

We also noted the patients maximum activity score (MAS) which is the maximum oxygen demanding activity the patient is still able to perform (as used in the HAP).

Patient’s notes and the renal IT data base (DiProton©) were accessed, with their permission, to gather a range of bio-chemical markers. Haemoglobin levels (Hb) were used to assess the extent of anaemia within individual patients. Bone markers were used to assess the patients for chronic kidney disease mineral bone disorders (CKD-MBD) (Lopatte 2013). The background comorbidities were correlated against the two activity scores.

Both scores were analysed and compared to a healthy age- and sex matched control group (n = 50).

### Statistical analysis

Data was analysed using the StatsDirect© programme. Kruskal–Wallis test and Mann–Whitney tests were used to analyse the mean scoring differences between the renal cohort and the healthy control. Kruskal–Wallis was also used to analyse the mean differences between the 5 kidney disease sub-groups (CKD, HHD, ICHD, PD, TX). Spearman’s Rank Correlation was used to assess the effect of blood biochemistry and co-morbidities on activity levels.

## Results

Within the renal cohort there was a wide range of primary diagnoses (Table 1). Ages ranged from 18–85 years old (mean age 60.82 ± 14.10). 39 patients were female and 61 male. Table 2 shows the distribution of patients between CKD and RRT and the number of patients receiving EPO therapy. Patients with CKD had been known to the renal service for an average of 51 ± 37.48 months (maximum 154 months, minimum 10 months) and patients on renal replacement therapy for an average of 49.54 ± 48.73 months (maximum 192 months, minimum 6 months).

**Table 1:** Primary diagnosis

Primary diagnosis	Number of patients (n = 100)
Polycystic kidney disease	15
Glomerulonephritis	15
Hypertension	14
Diabetic nephropathy	11
Pyelonephritis	11
IgA nephropathy	6
Unknown	8
Other	20

Each primary diagnosis and date of diagnosis was noted for every patient that filled out the questionnaire. A cohort with a wide range of primary diagnosis was preferable to show activity levels were not disease specific

**Table 2:** Patient characteristics

Group	Patient distribution in cohort (n = 100)	Number of patients on EPO (N = 45)
CKD stages 3–5	17	1
Home haemodialysis	17	11
In-centre haemodialysis	28	15
Peritoneal dialysis	17	16
Transplant recipients	21	2

100 patients filled out the questionnaire before their respective clinics. Patient records were used to assess their current EPO therapy status and used later for correlation

Results were compared to a healthy control group, ranging from 21 to 88 years old (mean age 59.34 ± 22.54 years old) with 24 females and 26 males within the sample.

### Maximum activity score

The maximum activity score in the renal population (CKD, RRT and transplant) ranged from 2 to 20, with the mean MAS being 12.53 ± 2.94. Comparing the CKD and individual RRT cohort groups to the healthy control population, a statistical significance was seen between the maximum activity score in the ICHD patients (11.4 ± 4.20) and the control (14.7 ± 4.24; p = 0.0083) (Table 3).

**Table 3:** Median and mean maximum activity scores

Sample sub-group	Median MAS (IQR)	Mean MAS (SD)	p value
CKD stages 3–5	13 (11–14)	12.2 (2.14)	0.0621
Home haemodialysis	13 (11–14)	12.5 (2.12)	0.1267
In-centre haemodialysis*	13 (8–14)	11.4 (4.20)	0.0083
Peritoneal dialysis	13 (13–14)	13.1 (1.17)	0.2929
Transplant recipients	13 (13–14)	13.8 (2.66)	0.4021
Control	14 (13–20)	14.76 (4.24)	–

Both median and mean maximum activity scores were calculated for each renal sub-group. Each sub-group was compared against the healthy control for statistical significance (p < 0.05)

* Statistically significant (p < 0.05) compared to the control group

### Total activity score

**Fig. 1:**
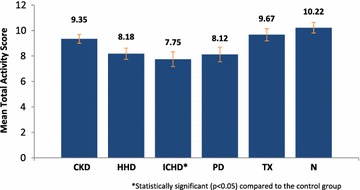
Mean total activity score. Graph to show the mean total activity scores within each of the renal sub-groups. *Statistical significance was reached comparing the ICHD patients to the control (p < 0.05)

The TAS within the renal population ranged from 2 to 14, with a mean score of 8.56 ± 2.43. There was significantly more activity seen in the control group (mean 10.22 ± 2.94) compared to ICHD (mean 7.75 ± 3.07) (p = 0.0188). No statistical significance was seen comparing the 5 sub-groups against each other (Table 4; Fig. 1).

**Table 4:** Median and mean total activity scores

Sample sub-group	Median TAS (IQR)	Mean TAS (SD)	p value
CKD stages 3–5	10 (9–10)	9.35 (1.46)	0.9828
Home haemodialysis	8 (7–9)	8.18 (1.81)	0.0535
In-centre haemodialysis*	8 (5–10)	7.75 (3.07)	0.0188
Peritoneal dialysis	8 (7–10)	8.12 (2.34)	0.0673
Transplant	9 (8–12)	9.67 (2.20)	0.7865
Control	10 (8–13)	10.22 (2.94)	–

Both median and mean total activity scores were calculated for each renal sub-group. Each sub-group was compared against the healthy control for statistical significance (p < 0.05)

* Statistically significant (p < 0.05) compared to the control group

### Activity loss

**Fig. 2:**
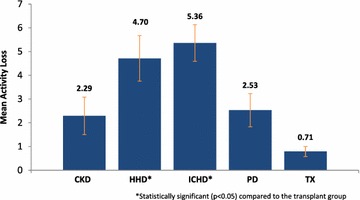
Mean activity loss score. Graph to show the mean activity loss per patient since their diagnosis/intervention within each renal sub-group. Statistical significance (p < 0.05) was reached within HHD and ICHD patients compared to the transplant group

The activity loss within the ICHD (5.36 ± 4.06, p = 0.0003) and the HHD (4.70 ± 3.96, p = 0.0038) was significantly higher when compared to the transplant patients (0.71 ± 0.96) (Table 5; Fig. 2).

**Table 5:** Median and mean activity loss scores

Sample sub-group	Median activity loss (IQR)	Mean activity loss (SD)	p value
CKD stages 3–5	1 (0–2)	2.29 (3.27)	0.6440
Home haemodialysis*	5 (1–7)	4.70 (3.96)	0.0038
In-centre haemodialysis*	6 (1–8)	5.36 (4.06)	0.0003
Peritoneal dialysis	2 (0–4)	2.53 (2.90)	0.1335
Transplant recipients	0 (0–1)	0.71 (0.96)	–

Both median and mean activity loss scores were calculated. Each sub-group was compared against the transplant group for statistical significance (p < 0.05)

* Statistically significant (p < 0.05) compared to the transplant group

### Erythropoietin

Mean haemoglobin levels for patients on EPO were lower (mean 107.5 g/L ± 11.84) when compared to those not on EPO (mean 125.4 g/L ± 15.0). Patients who were not on EPO therapy had a significantly higher level of activity (p = 0.013) and less activity loss when compared to patients on EPO therapy (p = 0.0024) (Tables 6, 7).

**Table 6:** Median and mean total activity scores for EPO versus no EPO therapy

Group	Median activity loss (IQR)	Mean activity loss (SD)	p value
EPO therapy	4 (1–7)	4.42 (3.88)	0.0024
No EPO therapy	1 (0–3)	2.33 (3.24)	

Median and mean total activity scores were calculated for patients currently receiving EPO therapy for their CKD-induced anaemia and compared to those who did not need EPO therapy

**Table 7:** Median and mean activity loss scores for EPO versus no EPO therapy

Group	Median TAS (IQR)	Mean TAS (SD)	p value
EPO therapy	8 (7–10)	7.84 (2.25)	0.013
No EPO therapy	9 (7–11)	9.15 (2.45)	

Median and mean activity loss scores were calculated for patients currently receiving EPO therapy for their CKD-induced anaemia and compared to those who did not require EPO therapy

Of the 21 transplant patients in the renal cohort only 2 patients were on EPO, with an average haemoglobin level of 135.1 g/L for the group.

### Blood biochemistry

**Fig. 3:**
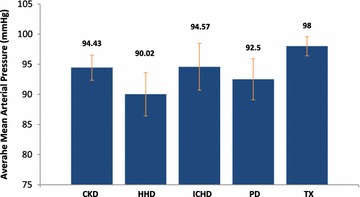
The average mean arterial pressure within each renal sub-group. Graph to show the differences in mean arterial pressure between the 5 renal sub-groups

No significant correlation was seen between calcium, phosphate or parathyroid hormone and the TAS of a patient. There was also no correlation with these parameters and the activity loss within the renal cohort. However significant positive correlation (r = 0.58, p = 0.0061) was seen between haemoglobin levels and TAS within the transplant group, illustrating that as the haemoglobin levels increased, so did their total activity levels (Table 8). Mean arterial pressure (MAP) showed significant positive (r = 0.54, p = 0.026) with the TAS in the PD group. This result shows that as the MAP decreases, the total activity of the patient decreases (Table 9). The average MAP for each subgroup can be seen in Fig. 3.

**Table 8:** Total activity and activity loss scores correlated with haemoglobin levels

Group	Score	Co-efficient	Co-efficient strength	p value
CKD stages 3–5	TAS	−0.38	Weak	0.13
Home haemodialysis	TAS	0.31	Weak	0.21
In-centre haemodialysis	TAS	0.23	Weak	0.24
Peritoneal dialysis	TAS	0.012	Very weak	0.96
Transplant recipients*	TAS	0.58	Moderate	0.0061
CKD stages 3–5	Activity loss	0.15	Very weak	0.55
Home haemodialysis	Activity loss	0.34	Weak	0.17
In-centre haemodialysis	Activity loss	−0.16	Very weak	0.41
Peritoneal dialysis	Activity loss	−0.33	Weak	0.19
Transplant recipients	Activity loss	−0.25	Weak	0.27

Table displaying the correlation co-efficient and p value for both activity scores against the haemoglobin levels for patients in each renal sub-group

*Statistically significant (p < 0.05) Spearman’s Rank Correlation between stated activity score and haemoglobin levels

**Table 9:** Total activity and activity loss scores correlated with mean arterial pressure

Group	Score	Co-efficient	Co-efficient strength	p value
CKD stages 3–5	TAS	0.39	Weak	0.12
Home haemodialysis	TAS	−0.11	Very weak	0.68
In-centre haemodialysis	TAS	0.32	Weak	0.097
Peritoneal dialysis*	TAS	0.54	Moderate	0.026
Transplant recipients	TAS	0.096	Very weak	0.67
CKD stages 3–5	Activity loss	0.095	Very weak	0.71
Home haemodialysis	Activity loss	−0.43	Moderate	0.098
In-centre haemodialysis	Activity loss	−0.22	Weak	0.24
Peritoneal dialysis	Activity loss	−0.18	Very weak	0.48
Transplant recipients	Activity loss	−0.22	Weak	0.33

Table displaying the correlation co-efficient and p value for both activity scores against the mean arterial pressures for patients in each renal sub-group

*Statistically significant (p < 0.05) Spearman’s Rank Correlation between stated activity score and mean arterial pressures

**Fig. 4:**
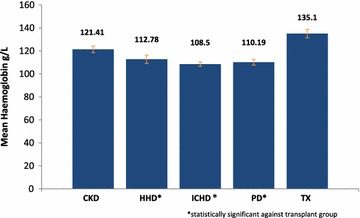
Mean haemoglobin levels within each renal sub-group. Graph to show the mean haemoglobin levels within each sub-group

A significant difference in haemoglobin levels was seen amongst the 3 dialysis subgroups compared to the transplant group (135.1 ± 17.23); HHD (112.78 ± 15.12, p = 0.0037), ICHD (108.50 ± 9.47, p < 0.0001) and PD (110.19 ± 10.08, p < 0.0001) (Fig. 4). There was no significant difference in haemoglobin levels between ICHD, PD or HHD patients. No significant correlation was noted between the haemoglobin levels of a patient and the number of activities they had lost (Table 8).

Surprisingly, there was no correlation seen between the number of co-morbidities a patient may have and their TAS or activity loss (Table 10).

**Table 10:** Total activity and activity loss scores correlated with the number of co-morbidities

Group	Score	Co-efficient	Co-efficient strength	p value
CKD stages 3–5	TAS	−0.31	Weak	0.23
Home haemodialysis	TAS	−0.08	Very weak	0.75
In-centre haemodialysis	TAS	−0.15	Very weak	0.43
Peritoneal dialysis	TAS	−0.30	Weak	0.24
Transplant	TAS	−0.43	Moderate	0.052
CKD stages 3–5	Activity loss	−0.18	Very weak	0.49
Home haemodialysis	Activity loss	0.19	Very weak	0.45
In-centre haemodialysis	Activity loss	0.015	Very weak	0.939
Peritoneal dialysis	Activity loss	0.0065	Very weak	0.98
Transplant	Activity loss	0.28	Weak	0.22

Table displaying the correlation co-efficient and p value for both activity scores against the number of co-morbidities for patients in each renal sub-group

## Discussion

The HAP has been used in several studies investigating the physical activity within the CKD population, however to our knowledge this is the first time an adapted version of the questionnaire has been used for the evaluation of physical activity in renal disease. This unique questionnaire saved valuable research time, allowing more patients to be included in the study.

We believe our results highlight the lack of activity seen within ICHD patients compared to the general population. Previous studies reported a decrease in physical activity throughout the entire CKD population (CKD, HHD, ICHD, PD) (Johansen et al. 2000; Painter and Johansen 2006). However within our study we found no significant difference between the CKD, HHD, TX and PD patients against the control (Table 4). This piece of research therefore favours home therapy over in-centre dialysis with regards the patient retaining their previous physical activity.

Total activity and activity loss scores were correlated with bio-chemical markers to assess if co-morbidities such as secondary hyperparathyroidism, anaemia and hypotension had any effect on physical activity in the different groups studied. No significant correlation was seen between the bio-chemical bone markers and total activity or activity loss scores. However these findings are contrary to current literature outcomes which suggest vitamin D deficiency and secondary hyperparathyroidism are associated with an overall poor physical function and activity level (Inderjeeth et al. 2000). However within this study we did not measure vitamin D directly but calcium, phosphate and PTH. Patients were not assessed for symptoms or signs related to CKD-MBD such as bone, joint and muscle pain either.

A significant positive correlation was seen within the TAS of the transplant group and their haemoglobin levels (Table 8). As the haemoglobin levels increased within this group, a significant increase in TAS was seen. Previous studies have looked at the association between CKD and anaemia with physical activity. Each variable was looked at separately and it was found that both CKD and anaemia independently reduced the physical activity of patients. When both these variables were analysed together, a significantly lower activity level was seen in these patients than those variables alone (Odden et al. 2004). Therefore we can conclude that anaemia and CKD will have an effect on the overall activity of a patient. As the patients within the transplant group have a significant increase in their kidney function and have an average Hb level above the cut off for anaemia, the overall activity will be higher within this group.

A significant correlation was also seen between the patient’s MAP and their physical activity within the dialysis group (Table 9). A significant positive correlation between the MAP and TAS showed that an increase in MAP was associated with an increase in activity. A significant negative correlation was also seen within this parameter which showed an increase in activity loss with a decreasing MAP. A potential explanation for this could be a state of chronic hypotension, a complication of dialysis seen in 5–10 % of patients (Cases and Coll 2002). The MAP in the study was comparable between the study groups however ICHD patients tend to drop their blood pressure during and post dialysis. Patients displaying symptoms of hypotension such as dizziness, weakness and fatigue, are less likely to engage in physical activity and may also have a lack of motivation towards exercise.

With a significant proportion of renal patients suffering from anaemia, patients often present with fatigue and breathlessness. This study found that patients on EPO therapy had lower levels of activity than those not on EPO therapy (Table 6). From our study it was also seen that patients on EPO therapy had a significantly greater activity loss than those not on EPO therapy (Table 7). A reason for this may be due to the lower haemoglobin levels seen within this group. This chronic state of anaemia will amplify symptoms, therefore causing them to engage in fewer activities.

In order to recruit enough patients for the study we had to create a short, concise questionnaire that would fully assess a patient’s physical activity whilst time efficient. We believe a key advantage to our questionnaire over the other questionnaires available is the length of time it takes to complete. Our questionnaire takes approximately 60 s to complete, whilst the HAP takes on average 5–10 min to complete (Bilek et al. 2005). This will save health care professionals valuable time which they can then use to educate their patients on the role and benefits physical activity can have within CKD. By taking key elements from the HAP to assess self-care, personal/household work, entertainment/social and personal exercise we were able to produce a 60 s questionnaire which enabled us to assess the patients physical activity and therefore their quality of life. With kidney disease increasing year after year in the UK and more pressure being put on the NHS’s resources, precious minutes saved by completing a shorter questionnaire could save the NHS both time and money, in an era in which both are in short supply.

A total of 100 patients filled out the questionnaire, with a control group of 50 healthy volunteers to compare the results to. This large number of patients strengthens the reliability of our results allowing significant trends to be seen within a particular group of patients. Terwee et al. (2010) reported that 100 patients is an ideal study size for evaluating the reliability of a scoring system as it ensures stability of the variance–covariance matrix.

Both of the questionnaires that were combined to develop our questionnaire are widely used within the UK and worldwide. The HAP scoring system has been shown to have a direct correlation with the physical activity of patients suffering from not only kidney disease, but other chronic conditions too (Robinson-Cohen et al. 2013). The definition of physical activity incorporates activity within the home, work and leisure time. Therefore the GPPAQ was applied to ask specific activities about the patient’s daily routine at work e.g. Sitting/standing most of the time. By combining two validated questionnaires, we felt this would strengthen our results.

Another advantage of this questionnaire is that activities that the patients never did were not taken into account during scoring; this meant that both the TAS and activity loss scores would not be affected by information that may be irrelevant to that particular patient.

As this was a questionnaire based research project, the validity of the findings may be affected due to the subjective nature of the results recorded. Patients for example may be inclined to over/under estimate the amount of activity they can do on a daily basis. A way to increase the validity of this study for future research may be to put in place some objective measurements such as pedometers. Inclusion of a partners/close contact opinion may also limit the bias nature of the results.

Within the published HAP questionnaire there are 94 questions for each patient to answer. This allows the assessor to gain a greater depth of knowledge on activities the patient is able and unable to do, which our questionnaire was only able to do to a certain extent. No significant difference was seen within the groups when comparing the MAS against each other. This we feel was due to the majority of the renal patients being able to achieve activity 13 (walking for less than 30 min, three times a week), however very few were able to achieve higher oxygen demanding activities than this (Additional file 1). By having more activities to select from, with a smaller energy demanding interval between them, a statistical significance may have been seen within this score.

This quick screening tool may also be very effective within the community. Community nurses on home visits can use this assessment tool to implement structured exercise plans within their high risk patients. This management plan can be tailored made to the individual patient, aiming to increase their physical activity within the particular area they may be lacking activity e.g. at work. As renal nurses have a lot of patient contact they are the ideal candidates to educate, coach and support a patient throughout their rehab period. The tool can then be used again in the future to assess if the patient’s physical activity level has increased or not.

## Conclusion

This study highlights the lack of activity in patients living with kidney disease. Even with the overwhelming evidence of the physical and psychological benefits of exercise within this high-risk group, there still appears to be inactivity present. We recommend further research be carried out on this quick, reliable tool to establish its use in clinics, dialysis centres and within the community to assess physical activity levels of patients. Tailor-made rehabilitation programmes can then be ‘prescribed’ to all patients suffering from kidney disease, preventing CVD and increasing their quality of life.

## Additional file

**Additional file 1.** Physical Activity Questionnaire for Renal Patients.

## References

[CR1] Bastone A, Moreira B, Vieira R, Kirkwood R (2013) Validation of the human activity profile questionnaire as a measure of physical activity levels in older community-dwelling women. J Aging Phys Act 22(3):348–56. doi:10.1123/japa.2013-0006 [cited 2014 Jul 7]. Available from: http://www.ncbi.nlm.nih.gov/pubmed/23917084 10.1123/JAPA.2012-0283 36724381

[CR2] Bilek LD, Venema DM, Camp KL, Lyden ER, Meza JL (2005) Evaluation of the human activity profile for use with persons with arthritis. Arthritis Rheum 53(5):756–63 [cited 2014 Jul 7]. Available from: http://www.ncbi.nlm.nih.gov/pubmed/16208665 10.1002/art.21455 16208665

[CR3] Cases A, Coll E (2002) Chronic hypotension in the dialysis patient. J Nephrol 15(4):331–5 [cited 2014 Jul 10]. Available from: http://www.ncbi.nlm.nih.gov/pubmed/12243360 12243360

[CR4] Davidson M, de Morton N (2007) A systematic review of the Human Activity Profile. Clin Rehabil 21(2):151–62 [cited 2014 Oct 25]. Available from: http://www.ncbi.nlm.nih.gov/pubmed/17264109 10.1177/0269215506069475 17264109

[CR5] Inderjeeth CA, Nicklason F, Al-Lahham Y, Greenaway TM, Jones G, Parameswaran VV et al (2000) Vitamin D deficiency and secondary hyperparathyroidism: clinical and biochemical associations in older non-institutionalised Southern Tasmanians. Aust NZ J Med 30(2):209–14 [cited 2014 Jul 13]. Available from: http://www.ncbi.nlm.nih.gov/pubmed/10833112 10.1111/j.1445-5994.2000.tb00809.x 10833112

[CR6] Johansen KL (2005) Exercise and chronic kidney disease: current recommendations. Sports Med 35(6):485–99 [cited 2014 Jul 6]. Available from: http://www.ncbi.nlm.nih.gov/pubmed/15974634 10.2165/00007256-200535060-00003 15974634

[CR7] Johansen KL, Chertow GM, Ng AV, Mulligan K, Carey S, Schoenfeld PY et al (2000) Physical activity levels in patients on hemodialysis and healthy sedentary controls. Kidney Int 57(6):2564–70 [cited 2014 May 24]. Available from: http://www.ncbi.nlm.nih.gov/pubmed/10844626 10.1046/j.1523-1755.2000.00116.x 10844626

[CR8] Johansen KL, Painter P, Kent-Braun JA, Ng AV, Carey S, Da Silva M et al (2001) Validation of questionnaires to estimate physical activity and functioning in end-stage renal disease. Kidney Int 59(3):1121–7 [cited 2014 Jul 7]. Available from: http://www.ncbi.nlm.nih.gov/pubmed/11231369 10.1046/j.1523-1755.2001.0590031121.x 11231369

[CR9] Lopatte G (2013) Myopathies associated with parathyroid disorders [cited 2014 Jul 3]. Available from: http://www.medlink.com/medlinkcontent.asp

[CR10] Meguid El Nahas A, Bello AK (2005) Chronic kidney disease: the global challenge. Lancet 365(9456):331–40 [cited 2014 Dec 13]. Available from: http://www.ncbi.nlm.nih.gov/pubmed/15664230 10.1016/S0140-6736(05)17789-7 15664230

[CR12] Odden MC, Whooley MA, Shlipak MG (2004) Association of chronic kidney disease and anemia with physical capacity: the heart and soul study. J Am Soc Nephrol 15(11):2908–15 [cited 2014 Jul 9]. Available from: http://www.pubmedcentral.nih.gov/articlerender.fcgi?artid=2776664&tool=pmcentrez&rendertype=abstract 10.1097/01.ASN.0000143743.78092.E3 PMC2776664 15504944

[CR13] Paffenbarger RS, Hyde RT, Wing AL, Lee IM, Jung DL, Kampert JB (1993) The association of changes in physical-activity level and other lifestyle characteristics with mortality among men. N Engl J Med 328(8):538–45 [cited 2014 May 28]. Available from: http://www.ncbi.nlm.nih.gov/pubmed/8426621 10.1056/NEJM199302253280804 8426621

[CR14] Painter P, Johansen KL (2006) Improving physical functioning: time to be a part of routine care. Am J Kidney Dis 48(1):167–70 [cited 2014 Jul 7]. Available from: http://www.ncbi.nlm.nih.gov/pubmed/16797401 10.1053/j.ajkd.2006.05.004 16797401

[CR15] Painter P, Marcus RL (2013) Assessing physical function and physical activity in patients with CKD. Clin J Am Soc Nephrol 8(5):861–72 [cited 2014 Oct 9]. Available from: http://www.ncbi.nlm.nih.gov/pubmed/23220421 10.2215/CJN.06590712 23220421

[CR16] Robinson-Cohen C, Littman AJ, Duncan GE, Roshanravan B, Ikizler TA, Himmelfarb J et al (2013) Assessment of physical activity in chronic kidney disease. J Ren Nutr 23(2):123–31 [cited 2014 Jun 10]. Available from: http://www.pubmedcentral.nih.gov/articlerender.fcgi?artid=3496802&tool=pmcentrez&rendertype=abstract 10.1053/j.jrn.2012.04.008 PMC3496802 22739659

[CR17] Sahlqvist S, Song Y, Bull F, Adams E, Preston J, Ogilvie D (2011) Effect of questionnaire length, personalisation and reminder type on response rate to a complex postal survey: randomised controlled trial. BMC Med Res Methodol 11:62 [cited 2015 Jul 21]. Available from: http://www.pubmedcentral.nih.gov/articlerender.fcgi?artid=3110121&tool=pmcentrez&rendertype=abstract 10.1186/1471-2288-11-62 PMC3110121 21548947

[CR18] Sarnak MJ (2003) Cardiovascular complications in chronic kidney disease. Am J Kidney Dis 41(5 Suppl):11–7 [cited 2014 Nov 3]. Available from: http://www.ncbi.nlm.nih.gov/pubmed/12776309 10.1016/s0272-6386(03)00372-x 12776309

[CR19] Steffen-Batey L, Nichaman MZ, Goff DC, Frankowski RF, Hanis CL, Ramsey DJ et al (2000) Change in level of physical activity and risk of all-cause mortality or reinfarction : the Corpus Christi Heart Project. Circulation 102(18):2204–9 [cited 2014 Jul 6]. Available from: http://circ.ahajournals.org/content/102/18/2204.full 10.1161/01.cir.102.18.2204 11056093

[CR501] Terwee CB, Mokkink LB, van Poppel MN, Chinapaw MJ, van Mechelen W, de Vet HC (2010) Qualitative attributes and measurement properties of physical activity questionnaires: a checklist. Sports Med 40(7):525–537. Available at: http://www.ncbi.nlm.nih.gov/pubmed/20545379 10.2165/11531370-000000000-00000 20545379

[CR11] (UK) NCC for N and SC (2008) The General Practice Physical Activity Questionnaire (GPPAQ). Royal College of Nursing (UK) [cited 2014 Nov 3]. Available from: http://www.ncbi.nlm.nih.gov/books/PMH0009982/

[CR20] Warburton DER, Nicol CW, Bredin SSD (2006) Health benefits of physical activity: the evidence. CMAJ 174(6):801–9 [cited 2014 May 26]. Available from: http://www.pubmedcentral.nih.gov/articlerender.fcgi?artid=1402378&tool=pmcentrez&rendertype=abstract 10.1503/cmaj.051351 PMC1402378 16534088

[CR21] Weir MR (2011) Recognizing the link between chronic kidney disease and cardiovascular disease. Am J Manag Care 17(Suppl 1):S396–402 [cited 2014 Jul 2]. Available from: http://www.ncbi.nlm.nih.gov/pubmed/22214474 22214474

