# Erratum to: Assessment of physical activity in patients with chronic kidney disease and renal replacement therapy

**DOI:** 10.1186/s40064-016-2529-2

**Published:** 2016-07-04

**Authors:** William S. G. Hayhurst, Aimun Ahmed

**Affiliations:** Renal Department, Royal Preston Hospital, Lancashire Teaching Hospitals NHS Foundation Trust, Preston, UK; Medical School, University of Manchester, Manchester, UK; Nephrology Department, Faculty of Medicine, Ain Shams University, Cairo, Egypt

## Erratum to: SpringerPlus (2015) 4:536 DOI 10.1186/s40064-015-1338-3

The original version of the article (Hayhurst and Ahmed 2015) did not include the second affiliation of Dr. Aimun Ahmed. This has now been included in this erratum.
