# Supplementary material for: Assessment of physical activity in patients with chronic kidney disease and renal replacement therapy
Source: Springerplus. 2015 Sep 21;4(1):536. doi: 10.1186/s40064-015-1338-3 (PMC4577500; doi:10.1186/s40064-015-1338-3)
Supplement: Supplementary file 1 — Additional file 1. Physical Activity Questionnaire for Renal Patients. [file 40064_2015_1338_MOESM1_ESM.docx]

**
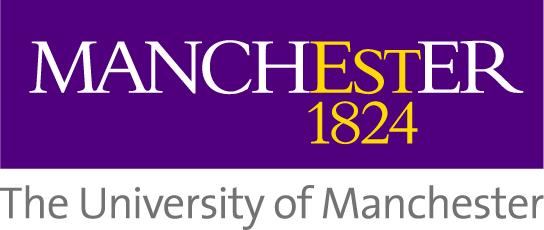
CKD ACTIVITY SCORE**

**NAME: D.O.B:**

**DATE: Type of Dialysis (if applicable):**

**Not doing this activity anymore since diagnosis/ starting RRT**

**Never did this activity**

**Still doing this activity**

**AT HOME:**

|  |  |  |
| --- | --- | --- |
|  |  |  |
|  |  |  |
|  |  |  |
|  |  |  |
|  |  |  |
|  |  |  |
|  |  |  |
|  |  |  |
|  |  |  |
|  |  |  |
|  |  |  |
|  |  |  |
|  |  |  |
|  |  |  |
|  |  |  |
|  |  |  |
|  |  |  |
|  |  |  |
|  |  |  |

1. **Get out of bed/chair without assistance.**
2. **Listen to the radio/watch Television……**
3. **Read the newspaper/book……………………**
4. **I spend most my time at work sitting…….**
5. **I get myself changed…………………………….**
6. **Shower/bath without assistance…………..**
7. **Make meals for yourself………………………**
8. **Clean the house…………………………………….**
9. **Do the laundry……………………………………..**
10. **I spend most my time at work standing..**
11. **Climb the stairs without assistance.........**
12. **My work involves definite physical effort ………………………………………………….**
13. **Walk for less than 30mins a day……………**
14. **Walk for more than 30mins a day…………**
15. **Swimming 25 metres non-stop……………..**
16. **Cycle for less than a mile………………………**
17. **Cycle for more than a mile…………………..**
18. **Running for less than 1 mile…………………**
19. **Running for more than 1 mile………………**
20. **Play sport eg. Football, tennis………………**
